# The Effects of Amino Acid Composition of Glutamine-Rich Domains on Amyloid Formation and Fragmentation

**DOI:** 10.1371/journal.pone.0046458

**Published:** 2012-10-10

**Authors:** Alexander I. Alexandrov, Alla B. Polyanskaya, Genrikh V. Serpionov, Michael D. Ter-Avanesyan, Vitaly V. Kushnirov

**Affiliations:** A. N. Bach Institute of Biochemistry of the Russian Academy of Sciences, Moscow, Russia; University of Maryland, United States of America

## Abstract

Fragmentation of amyloid polymers by the chaperone Hsp104 allows them to propagate as prions in yeast. The factors which determine the frequency of fragmentation are unclear, though it is often presumed to depend on the physical strength of prion polymers. Proteins with long polyglutamine stretches represent a tractable model for revealing sequence elements required for polymer fragmentation in yeast, since they form poorly fragmented amyloids. Here we show that interspersion of polyglutamine stretches with various amino acid residues differentially affects the *in vivo* formation and fragmentation of the respective amyloids. Aromatic residues tyrosine, tryptophan and phenylalanine strongly stimulated polymer fragmentation, leading to the appearance of oligomers as small as dimers. Alanine, methionine, cysteine, serine, threonine and histidine also enhanced fragmentation, while charged residues, proline, glycine and leucine inhibited polymerization. Our data indicate that fragmentation frequency primarily depends on the recognition of fragmentation-promoting residues by Hsp104 and/or its co-chaperones, rather than on the physical stability of polymers. This suggests that differential exposure of such residues to chaperones defines prion variant-specific differences in polymer fragmentation efficiency.

## Introduction

Some proteins can undergo non-covalent polymerization coupled with conformational rearrangement, resulting in the formation of amyloid fibrils with regular cross-β-sheet structure. Such fibrils tend to stick together, forming intra- or extracellular amyloid aggregates. Amyloid formation causes about 30 diseases, also called amyloidoses, many of which are neurodegenerative, including Alzheimer, Parkinson, and Huntington diseases [1], [2]. Amyloidoses are thought to be noninfectious, except for the prion diseases related to the PrP protein, which include Creutzfeldt-Jacob disease, sheep scrapie, and other transmissible spongiform encephalopathies.

Prions were also found in fungi, mostly in the yeast *Saccharomyces cerevisiae*, where they define various heritable phenotypes. Yeast prions represent a convenient model for studying the basic properties of prions. Among them, [*PSI* ^+^] is probably the best studied. This prion is related to the heritable polymerization of translation termination factor eRF3, also called Sup35, which reduces efficiency of translation termination resulting in a nonsense-suppressor phenotype. The *S. cerevisiae* Sup35 protein consists of three domains [3], [4]. Its amino-terminal N domain (amino acid residues (aa) 1–123), also called prion domain (PrD), is responsible for the prion properties of the protein, being necessary for its polymerization both *in vivo* [5] and *in vitro* [6]. The Sup35 PrD can be split into two areas, one of which (aa 1–40) is especially rich in glutamine (Q) and asparagine (N), while another (aa 41–123) contains five and a half imperfect oligopeptide repeats and has a high proportion of tyrosine, glycine and proline. The carboxyl-terminal C domain of Sup35 (aa 254–685) is responsible for the translation termination activity of the protein [3]. The Sup35 middle M domain (aa 124–253) probably serves as a spacer between the N and C domains and contains a binding site (aa 129–148) for the Hsp104 chaperone [7]. The propagation of [*PSI* ^+^] requires Hsp104 [8], which fragments Sup35 polymers, thus multiplying Sup35 prion particles [9], [10]. Similarly to mammalian prions, yeast prions, and [*PSI* ^+^] in particular, can exist in different phenotypic variants. “Strong” [*PSI* ^+^] variants, as compared to “weak” ones, show more efficient nonsense suppression and higher mitotic stability. These phenotypic differences are defined by variation in the fragmentation frequency of Sup35 polymers, which is higher in strong [*PSI* ^+^] variants [10]–[12]. However, the structural elements of prion polymers, which determine their fragmentation frequency, remain unclear.

Amyloid and prion polymers are distinguished by high physical strength. In contrast to other large protein structures, they are insoluble at room temperature in strong ionic detergents such as sodium lauroyl sarcosinate (Sarcosyl) and sodium dodecyl sulphate (SDS), allowing to analyze the size of these polymers using agarose electrophoresis in the presence of these detergents (SDD-AGE) [10]. The fragmentation frequency can be judged by the size of prion polymers, with one being inversely proportional to the other. Remarkably, this dependence is unambiguous, i.e. the size should not depend on other parameters, such as rate constants for polymerization and prion synthesis [11], [13].

The prionogenic properties of Sup35 and most other yeast proteins rely on their Q/N-rich PrDs, which makes them similar to amyloidogenic proteins with expanded polyglutamine (polyQ) domains, such as the human huntingtin protein. However, in contrast to yeast prions, polymers of polyQ proteins are poorly fragmented in yeast and have a large size [13].

Chaperones are known to recognize misfolded proteins by binding to exposed hydrophobic residues [14], [15], and so we proposed that the frequency of amyloid polymer fragmentation mainly depends on recognition of such residues by Hsp104 and/or its co-chaperones. In agreement with this, insertion of tyrosine residues into polyQ stretches strongly decreased the size of SDS-resistant polymers, indicating that polymer fragmentation was greatly increased [13]. Another factor which is thought to influence the fragmentation of amyloid polymers by chaperones is physical stability of polymers. This assumption stems from the observation that the better fragmentation of strong [*PSI* ^+^] Sup35 polymers correlates with their higher fragility [10], [11], [16]. The fragility of these polymers can be related to observations that in them a smaller portion of the PrD is tightly packed into the amyloid core.

Here, we studied the effects of various amino acid residues inserted into polyQ stretches on amyloid formation and fragmentation in yeast. The most efficient fragmentation was caused by insertion of aromatic residues tyrosine, tryptophan and phenylalanine, while strongly hydrophobic residues valine and isoleucine did not promote fragmentation. Our data also show that the size of amyloid polymers does not always correlate with their thermal stability, indicating that physical stability of polymers does not define the susceptibility to fragmentation.

## Results

### PolyQX proteins and their ability to polymerize

We created a set of multicopy plasmids encoding various poly(QQQXQ) domains, where X is any amino acid residue (Table 1), fused to Sup35 MC domains. These proteins are further referred to as nQX, where n is the length of the polyQX stretch (such as 76QY, which has a 76 aa stretch of polyglutamine with tyrosine interspersions). Lysine and aspartic acid were not included, since we expected them to prevent polymer formation similarly to two other charged residues, arginine and glutamic acid (see below). Despite these plasmids being multicopy, they provided only 3-fold overproduction of the encoded proteins as compared to wild type Sup35 [13]).

**Table 1:** Appearance and size of polymers of QX proteins.

Group	I			II						III					IV			
Residue	Y	W	F	H	A	S	T	C	M	Q	N	I	V	G	R	E	L	P
[*PIN* ^+^]	+	+	+	+	+	+	+	+	+	+	+	+	+	+/−	−	−	−	−
Δ*rnq1*	+	+	+	+/−	−	+	+	+	+	+*	+*	+	+	−				
Q85 [*PIN* ^+^]															+	+	+	−
Polymer size	Small			Medium						Large					Large			

(+) denotes presence of SDS-resistant polymers, (−), polymers were absent, (+/−), low amount of polymers,

(*) , delayed emergence of polymers.

**Figure 1:**
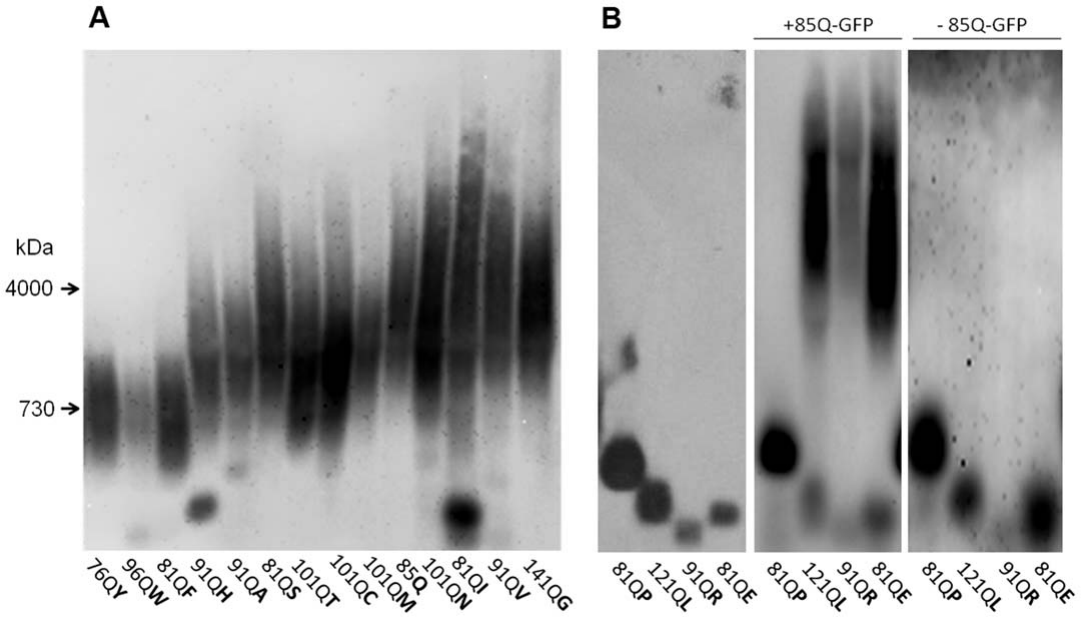
Polymerization of polyQX proteins in the presence of [*PIN* ^+^]. Lysates of 74-D694/ΔS35 [*PIN* ^+^] cells producing indicated QX proteins were analyzed by SDD-AGE. (A) Most QX proteins readily form SDS-insoluble polymers of varying size. Staining with anti-S35NM antibodies. (B) Polymerization of QX proteins, which did not polymerize in a [*PIN* ^+^] background on their own, in the presence of 85Q-GFP polymers and after loss of the plasmid encoding 85Q-GFP. Staining with anti-Sup35C.

The plasmids encoding polyQX proteins were introduced into cells of the 74-D694ΔS35 [*PIN* ^+^] and *Δrnq1* strains which lacked the chromosomal *SUP35* gene, but contained a plasmid encoding the C-domain of Sup35 to support viability. The ability of polyQX proteins to polymerize was assayed after the Sup35C-encoding plasmid was lost. The [*PIN* ^+^] determinant related to the Rnq1 protein [17] was present to facilitate the *de novo* appearance of polyQX polymers, which were detected using SDD-AGE (Figure 1).

**Figure 2:**
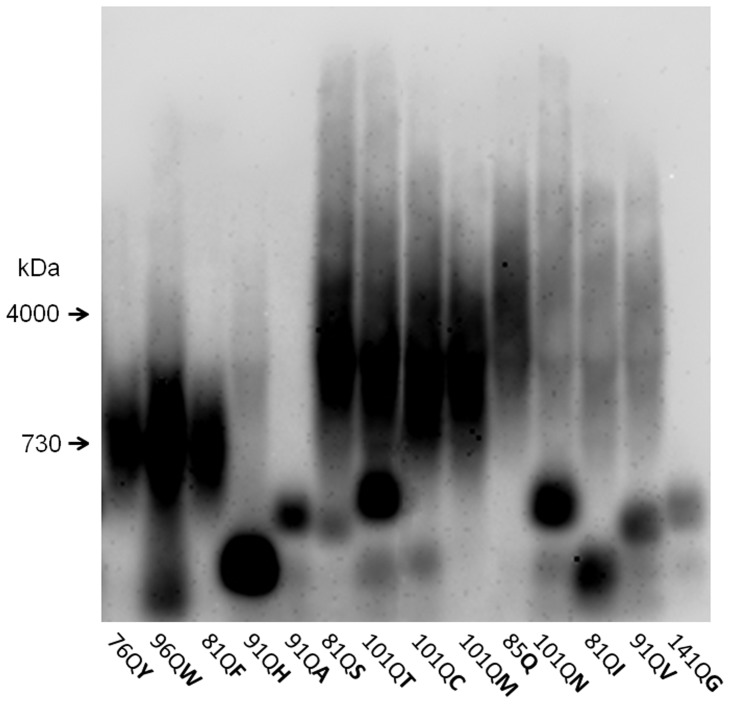
Polymerization of polyQX proteins in the absence of [*PIN* ^+^]. Lysates of 74-D694/ΔS35 Δ*RNQ1* cells producing QX proteins of different length were analyzed by SDD-AGE. Cells were grown for 20 generations after obtaining transformants. Staining with anti-S35NM antibodies.

All the amino acids were assigned into four groups according to their effects on amyloid formation and fragmentation. In the presence of [*PIN* ^+^], most polyQX proteins efficiently formed SDS-insoluble polymers (Figure 1A), except for QR, QE, QP and QL (Figure 1B). 101QG polymerized poorly though a longer polyQG protein, 141QG, showed efficient polymerization (Figure S1). In a *Δrnq1* background (Figure 2), most QX proteins, with the exception of 91QA and 141QG, formed noticeable amounts of SDS-resistant polymers. Notably, polymers of 85Q, 101QN and 91QH proteins appeared with a delay, i.e. they were not observed in fresh transformants, but appeared after 20 additional generations (Figures 2 and S2). The polyQR, QE, QP and QL proteins did not form SDS-resistant polymers in [*PIN* ^+^] cells. Polymers of these proteins were not observed even when the cells were plated onto adenine-omission medium selective for cells with decreased levels of soluble polyQX-Sup35MC, which can be caused by polymerization (data not shown). To determine whether these proteins could polymerize, their *in vivo* polymerization was seeded with 85Q-GFP (Figure 1B), presuming that polyQ polymers may represent better seeds than Rnq1 polymers. In the presence of 85Q-GFP polymers, 91QR, 81QE and 121QL proteins formed noticeable amounts of polymers, while 81QP did not. The seeded polymers could not propagate on their own, since loss of the plasmid encoding 85Q seeds resulted in their disappearance. Thus, polyQR, polyQE and polyQL cannot form polymers *in vivo* on their own, but can do so in a complex with polyQ.

### Effects of amino acid insertions on the size of the polyQ-based polymers

**Figure 3:**
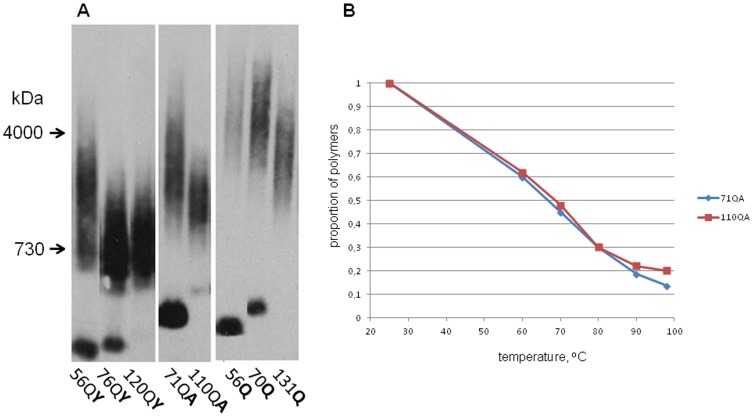
Polyglutamine domain length influences polymer size and not polymer stability. (A) Lysates of 74-D694/ΔS35 [*PIN* ^+^] cells producing QX proteins of different length were analyzed by SDD-AGE. Staining with anti-S35NM antibodies. (B) The lysates of 74-D694/ΔS35 [*PIN* ^+^] cells producing QA71 and QA110 proteins were incubated at different temperatures in the presence of sample buffer containing 2% SDS and analyzed by SDD-AGE. The thermal denaturation curves were obtained by densitometric analysis of the stained blot images.

The cloning strategy used in this work allowed us to obtain polyQX sequences of different length in a single round of cloning, but obtaining polyQX of identical size was complicated. This prompted us to assess whether polymer size significantly depends on the length of the polyQX stretch. Polymers showed a small decrease in size with increase of the polyQX length (Figure 3A). This allowed us to use proteins with a polyQX length in the range of 76–101 residues for studying fragmentation efficiency, since in this range the effect of polyQX length on polymer size was modest compared to that of the inserted amino acid residues.

Insertion of tyrosines, tryptophanes, or phenylalanines (group I) resulted in formation of the smallest polymers. Medium-sized polymers were formed by polyQ with alanine, methionine, cysteine, serine, threonine and histidine insertions (group II), and the largest polymers, similar in size to polyQ polymers, corresponded to insertion of asparagines, valines, isoleucines and glycines (group III) (Figure 1A, 2). Glycine could be attributed to both groups III and IV, since it did not stimulate fragmentation and significantly inhibited polymerization. Intensity plots with molecular weight references are presented in Figure S3.

Due to the presence of the *ade1-14* nonsense mutation in the used strain, it was possible to determine the nonsense suppressor phenotypes of cells producing QX proteins (Figure S4). The phenotypes, with some exceptions, showed the expected correlation with polymer size (See Supplementary Note S1 for details), i.e. cells with smaller polymers showed stronger suppressor phenotypes.

**Figure 4:**
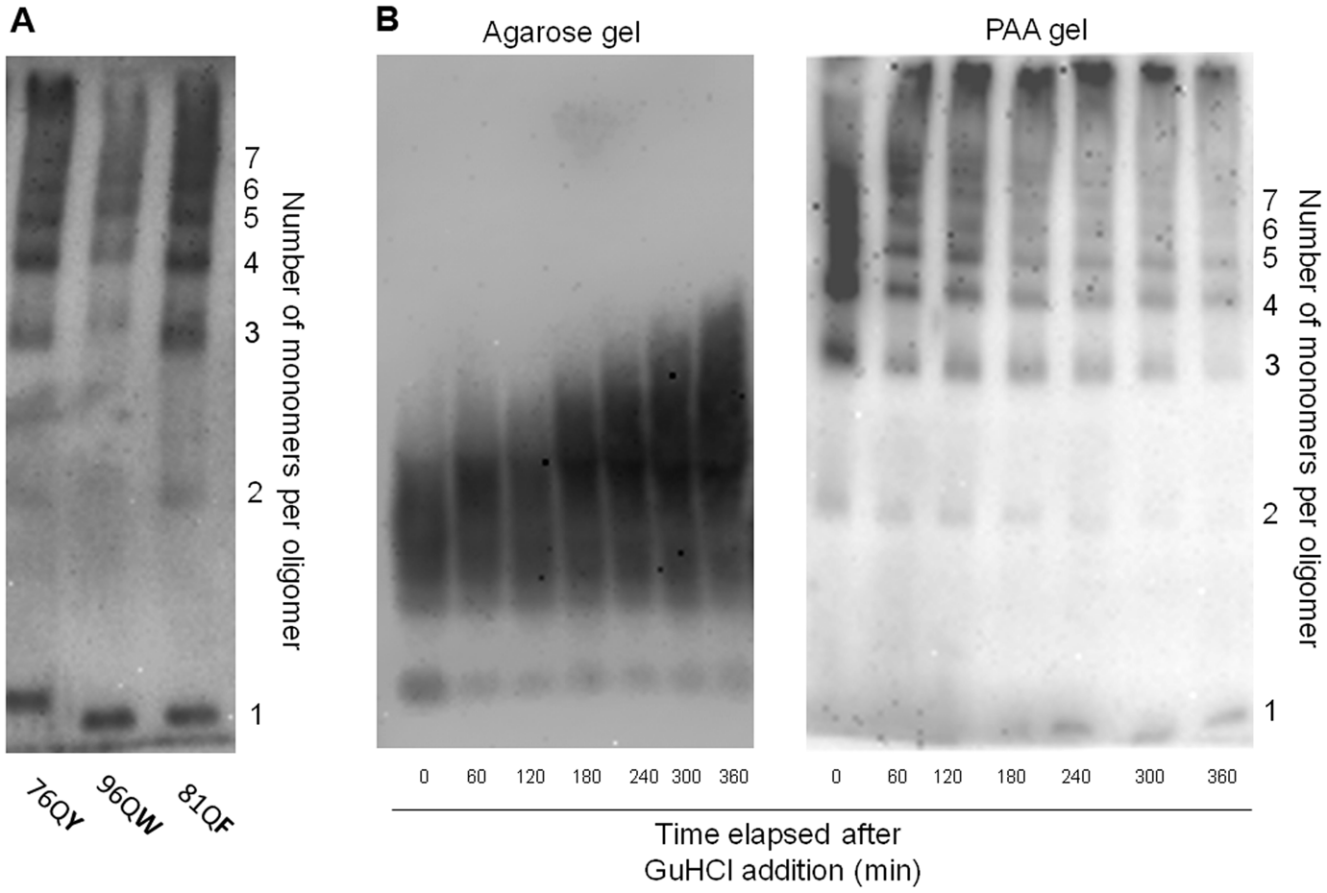
Small SDS-insoluble polymers of QX proteins. (A) Lysates of 74-D694/ΔS35 [*PIN* ^+^] Δ*prb1* cells producing 76QY, 96QW and 81QF were analyzed by SDS-PAGE without boiling the samples using large pore 5% gel. (B) 74-D694/ΔS35 [*PIN* ^+^] Δ*prb1* cells producing 76QY were grown for different periods of time in the presence of GuHCl (3 mM), and then their lysates were analyzed by SDD-AGE (left panel) and SDS-PAGE without boiling the samples (large pore 5% gel) (right panel).

The polymers formed by 76QY, 96QW and 81QF proteins were the smallest, which allowed us to determine their exact size using SDS-PAGE with large-pore 5% gel and unboiled samples. Polymers from wild-type strains exhibited some degradation and smearing (Figure S5), but deletion of the *PRB1* gene, which encodes the vacuolar proteinase B, dramatically reduced this effect and allowed to observe them as discrete bands. 76QY, 81QF and 96QW polymers were distributed in a ladder (Figure 4A) according to the number of monomers per polymer, with the smallest species apparently representing dimers. Importantly, the observed polymers were products of Hsp104 fragmentation activity, since they were able to increase in size upon growth of cells in the presence of Hsp104 inhibitor guanidine hydrochloride [13] (Figure 4B) and eventually did not enter the polyacrylamide gel.

### The size of amyloid polymers does not always correlate with their thermal stability

Next, we decided to test whether physical stability of polymers determines their fragmentation efficiency. Since the fragility of relatively small *ex vivo* polymers is difficult to determine, we measured their thermal stability in the presence of SDS, which was shown to correlate with physical stability [11], [16].

It can be expected that longer polyQX stretches form amyloids with larger and stronger cores, which should be fragmented less efficiently, thus yielding larger polymers. In contrast, we observed (Figure 3A) that the size of polymers decreased with increasing polyQX length. This decrease was moderate, but highly reproducible. The thermal stability of polymers of two polyQA proteins, 71QA and 110QA, was similar (Figure 3B), despite the 110QA polymers being smaller (Figure 3A).

**Figure 5:**
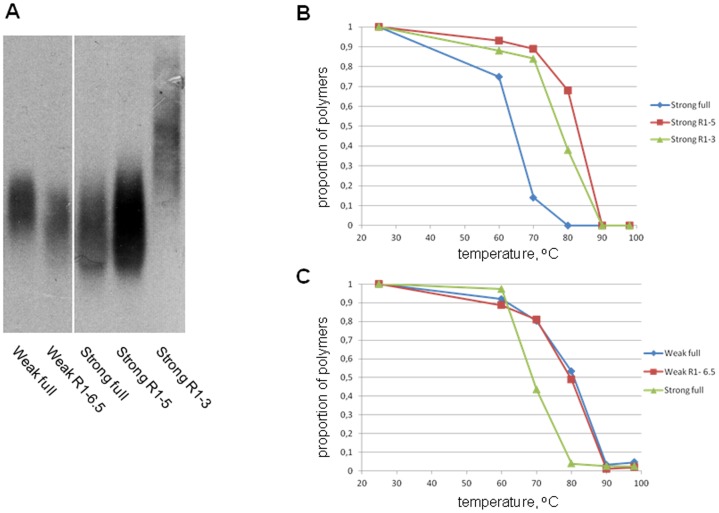
Thermal stability of prion polymers of full-size and truncated Sup35. (A) The lysates of [*PSI* ^+^] yeast cells producing full-sized or truncated Sup35 were analyzed by SDD-AGE. (B, C) Lysates were heated in the presence of sample buffer containing 2% SDS at different temperatures and analyzed by SDD-AGE. The thermal denaturation curves were derived from densitometric analysis of the stained blot images.

The lack of correlation between the size and stability of polymers was also observed for Sup35 prion polymers. It was previously shown that strong [*PSI* ^+^] variants can be transmitted to and maintained by truncated versions of Sup35, in which some of the C-terminal PrD repeats were removed. Such transmission resulted in increased size of Sup35 polymers and weakened [*PSI* ^+^] phenotype. However, the variant-specific fold appeared to be preserved, since transmission of [*PSI* ^+^] back to wild type Sup35 restored the strong [*PSI* ^+^] phenotype [18]. We studied the changes in the size and thermal stability of prion polymers of strong [*PSI* ^+^] after its transmission from full-length Sup35 to Sup35 with PrD containing either five (Sup35R1-5; Δ94–112 aa) or three (Sup35R1-3; Δ75–112 aa) oligopeptide repeats. [*PSI* ^+^] transmission to both Sup35R1-5 and Sup35R1-3 proteins was accompanied by a significant increase in the polymer thermal stability (Figure 5B), while increase in the polymer size was observed only for Sup35R1-3 (Figure 5A).

Sup35 polymers of strong [*PSI* ^+^] variants are usually significantly smaller than polymers of weak variants, which reflects their higher fragmentation frequency. However, with the [*PSI* ^+^] variants used in this work, the “strong” Sup35 polymers were only slightly smaller than the “weak” ones, despite showing a significant difference in their thermal stability (Figure 5C). Furthermore, the same weak [*PSI* ^+^] maintained by a truncated version of Sup35 (Sup35R1-6.5; Δ102–112 aa) resulted in polymers which were identical in size to the Sup35 polymers of strong [*PSI* ^+^], but had the same high thermal stability as weak [*PSI* ^+^] polymers.

The increase of polymer thermal stability upon [*PSI* ^+^] transmission from Sup35 to Sup35R1-3 was reproduced for another strong [*PSI* ^+^] variant (Figure S6). Intriguingly, this suggests that, despite PrD truncation, a longer region of this domain was tightly packed into the amyloid core than in the original full-length PrD. This may indicate the existence of a region which somehow restricts the extent of PrD packaging into the amyloid core.

## Discussion

### Staggered structure of polyQ/QX polymers

**Figure 6:**
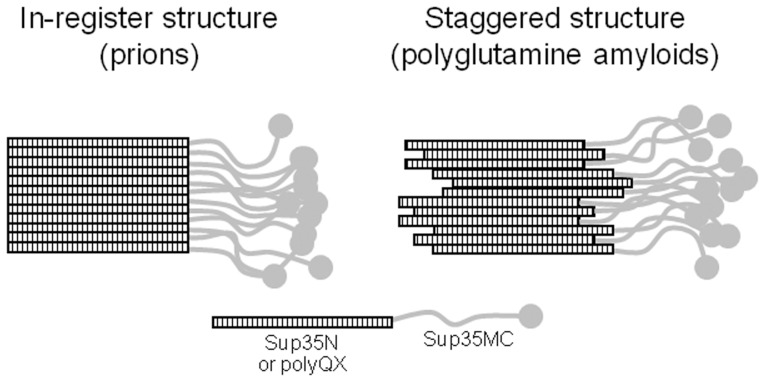
Staggered and fixed structures of amyloid polymers. Schematic representation of amyloid polymers with a fixed fold, as proposed for yeast prion polymers, and a staggered fold, as proposed for polyQX amyloids.

In prion polymers the adjacent molecules are located “in-register” [19], [20], while polyQ is a uniform sequence, which allows establishing the same intermolecular interactions out-of-register. Thus, polyQ and polyQX amyloids are likely to have a staggered structure, in which the length of intermolecular contacts varies (Figure 6). As can be expected due to such variation, we observed that the melting of polyQA and polyQY polymers occurs more gradually than the melting of prion Sup35 polymers (Figures 3B, 5 and S7).

The staggered structure of polyQ polymers allows using them as a model for studying the effects of various amino acids on fragmentation. In contrast, fragmentation of prions depends not only on the PrD sequence, but also, and probably to a greater extent, on the prion variant. Unlike prions, polyQX amyloids are likely to form no stable variants, because the structural information should be copied incompletely along a polymer due to staggered relative location of neighboring polyQX domains. Indeed we found no variation of polymer size among transformants bearing the same polyQX construct. In addition, as we observed (Figure 5), yeast prion polymers can undergo significant changes in the size of their amyloid cores in response to manipulations with their PrD sequence. It should be noted that the fragmentation frequency should mainly depend on the size of either the amyloid core or of the exposed region, while the particular variant-specific folding details are likely to be insignificant. In the staggered polyQX polymers both these sizes should randomly vary along a fibril, but their averaged values should not depend on either the nature of the inserted amino acids or the possible variation of folding of the amyloid core.

### Effects of amino acid insertions on polyQX amyloid formation and fragmentation

Earlier we demonstrated that interspersing polyQ with tyrosines dramatically increases the frequency of polymer fragmentation [13]. This supported our hypothesis that the fragmentation of amyloid polymers, performed by Hsp104, requires their recognition by this and/or other chaperones as misfolded protein structures, and that hydrophobic residues can act as signs of misfolding. Here, we further tested this hypothesis by studying the effects of incorporation of other amino acid residues into polyQ.

By their effect on polyQ amyloid formation and size, inserted amino acid residues were divided into four groups. Most of the residues (groups I–III) did not interfere with amyloid formation, though some residues interfered with polymerization in the *Δrnq1* cells, while others accelerated appearance of polymers (Figures 2 and S2 and Table 2). Insertion of three aromatic residues, representing the first group (tyrosine, tryptophan and phenylalanine) resulted in appearance of the smallest polymers. They can be considered as providing the highest frequency of polymer fragmentation, since according to the quantitative model of prion formation, the polymer size is inversely related to the fragmentation frequency and, furthermore, is defined solely by it [11], [13]. The most abundant particles of 76QY, 96QW and 81QF represented tetra- to hexamers, while the smallest species were dimers, which indicates that even dimers can support amyloid structure (Figure 4). For comparison, [*PSI* ^+^] particles usually contain 10 to 50 Sup35 molecules [10].

**Table 2:** Plasmids used in this study.

Plasmid	Short name	Interspersing aa
pSBSE	-	-
pSBSE-50Q	50Q	-
pSBSE-70Q	70Q	-
pSBSE-85Q	85Q	-
pSBSE-131Q	131Q	-
pSBSE-56QY	56QY	tyrosine
pSBSE-76QY	76QY	tyrosine
pSBSE-120QY	120QY	tyrosine
pSBSE-71QA	71QA	alanine
pSBSE-91QA	91QA	alanine
pSBSE-110QA	110QA	alanine
pSBSE-96QW	96QW	tryptophan
pSBSE-91QV	91QV	valine
pSBSE-81QF	81QF	phenylalanine
pSBSE-91QH	91QH	histidine
pSBSE-101QT	101QT	threonine
pSBSE-81QS	81QS	serine
pSBSE-101QC	101QC	cysteine
pSBSE-101QM	101QM	methionine
pSBSE-101QN	101QN	asparagine
pSBSE-81QI	81QI	isoleucine
pSBSE-81QP	81QP	proline
pSBSE-81QR	81QR	arginine
pSBSE-91QE	91QE	glutamic acid
pSBSE-121QL	121QL	leucine
pSBSE-101QG	101QG	glycine
pSBSE-141QG	141QG	glycine
YEplac181-85Q-M-GFP	85Q-GFP	-
pRS315-Sup35C	-	-

The second group includes six residues of different nature, alanine, cysteine, methionine, serine, threonine and histidine, which caused intermediate stimulation of fragmentation. Alanine, cysteine and methionine are weakly hydrophobic, while serine and threonine are polar and histidine is weakly charged. Residues of the third group did not stimulate fragmentation. They included asparagine, glycine, and, surprisingly, strongly hydrophobic isoleucine and valine.

Residues of the fourth group (arginine, glutamic acid, proline and leucine) prevented polyQX polymerization. Glycine also inhibited polymerization, albeit incompletely. 91QR, 81QE and 121QL were able to polymerize *in vivo* in the presence of Q85-GFP polymers. Most probably, 91QR and 81QE cannot polymerize due to repulsion of charged chains, but can do so, being interleaved with 85Q molecules. The inability of 121QL to polymerize on its own is more difficult to explain, especially taking into account the efficient polymerization of polyQV and polyQI proteins containing two other strongly hydrophobic amino acids. 81QP did not form SDS-resistant polymers in the presence of Q85-GFP, which is not surprising, since proline is known as a beta-sheet breaker.

Thus, it is possible to conclude that efficient fragmentation is caused by aromatic, rather than simply hydrophobic residues. The strongest hydrophobic residues either did not affect fragmentation (isoleucine and valine), or interfered with polymerization (leucine). Other residues stimulating fragmentation were of different nature, only half of them being hydrophobic.

### Factors affecting polymer fragmentation

The factors which may limit the frequency of amyloid fragmentation can be assigned to two stages of this process. At the first stage Hsp104 with the help of other chaperones recognizes a protein molecule in a polymer and binds to it. At the second, this molecule is threaded through the central pore of the ring-shaped Hsp104 hexamer, which results in fragmentation of the polymer.

The dependence of the frequency of polymer fragmentation on the efficiency of amyloid recognition by Hsp104 and/or its partner chaperones was shown by several groups. Removal of the Hsp104 binding site located within the Sup35 M domain (aa 129–148) noticeably decreased fragmentation of Sup35 prion polymers and weakened the [*PSI* ^+^] phenotype [7]. More profound decrease, leading to the [*PSI* ^+^] loss, was observed upon depletion of the Sis1 chaperone or the Ssa chaperones, all of which are thought to facilitate the Hsp104 binding to a target [12], [21]–[23]. This allows thinking that Sis1/Ssa play a key role in recognition of prion polymers, while recognition by Hsp104 is auxiliary. If so, the greatly improved fragmentation of some polyQX polymers observed here most likely was also due to recognition of the polyQX stretches by the Sis1/Ssa chaperones. Importantly, such recognition did not rely on any specific sequences, since polyQX stretches are not found in natural prions. The “scrambled” Sup35N domains were also prone to fragmentation [24], despite lacking sequences identical to wild-type Sup35 PrD. Thus, it can be assumed that prion recognition by chaperones depends mainly on amino acid composition of prionogenic domains and our data support our original assumption that amino acid residues act as individual recognition elements.

After binding, Hsp104 has to extract the bound molecule out of a polymer. The efficiency of this process could be affected by polymer stability. This is due to a specific property of Hsp104, which is absent in related proteins such as ClpC: it can stop unfolding a protein and dissociate when encountering a properly folded globular domain [25]. Since the amyloid core of a polymer is also a robust structure, it could serve as a signal for dissociation of Hsp104.

An additional possible factor is the ability of Hsp104 to grip the bound polypeptide strongly enough to apply a sufficient threading force. This interaction was shown to depend on two tyrosine residues of Hsp104 exposed into its inner cavity. Substitution of these residues for alanine caused backsliding of the threaded chain, while replacing them with other aromatic residues, phenylalanine or tryptophan, only moderately decreased the threading efficiency [26], [27]. Also, [*PSI* ^+^] can be propagated, albeit less efficiently, by Y662F and Y662W mutants of Hsp104 [28] On the other hand, the presence of aromatic residues in the substrate could also be important for efficient threading. Such residues were shown to be important for the substrate interaction with Hsp104: the affinity of a model peptide p370 (KLFSDDVFEREYA) to Hsp104 strongly depends on its aromatic residues [7], [29]. So, the observed high efficiency of aromatic residues in promoting amyloid polymer fragmentation may partly rely on facilitation of polypeptide threading.

**Figure 7:**
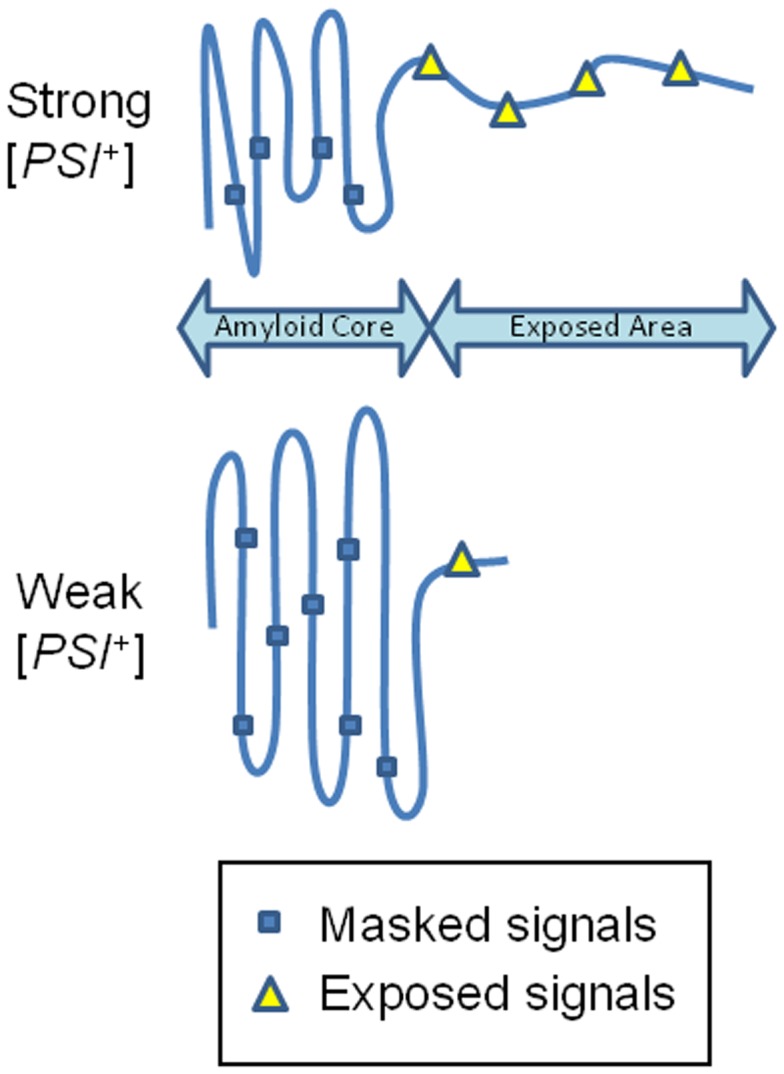
Schematic model of the structure of the Sup35 prion domain. Fragmentation-promoting residues (blue rectangles), such as tyrosine, are hidden inside the amyloid core structure and cannot act as recognition signals for chaperones. Residues in the exposed area (yellow triangles) are available and can thus facilitate fragmentation.

The differences in the fragmentation efficiency of “strong” and “weak” [*PSI* ^+^] polymers are easily explained by either the determining role of fiber fragility or chaperone recognition. In “strong” polymers a shorter region of the prion domain is involved in amyloid core, and so this core is more fragile. In the same polymers, a longer part of the N domain is not packed into the core and can be better recognized by fragmenting chaperones (Figure 7). Our data indicate that the role of the exposed region is more critical. Firstly, we observed that elongation of polyQX tracts improves fragmentation of respective polymers. In the staggered out-of register structure of polyQX polymers, an increase in polyQX length should increase the average size of both the amyloid core and the unfolded polyQX regions, thus increasing polymer strength and presumably improving chaperone recognition. The observed increase in fragmentation frequency indicates that the effect of chaperone recognition is much more significant than that of fragility. Secondly, the lack of correlation between the physical stability of Sup35 polymers and the frequency of their fragmentation also indicates that mechanical strength was not a major factor defining the efficiency of amyloid fragmentation.

### Occurrence of fragmentation-promoting amino acids in yeast prion domains

**Figure 8:**
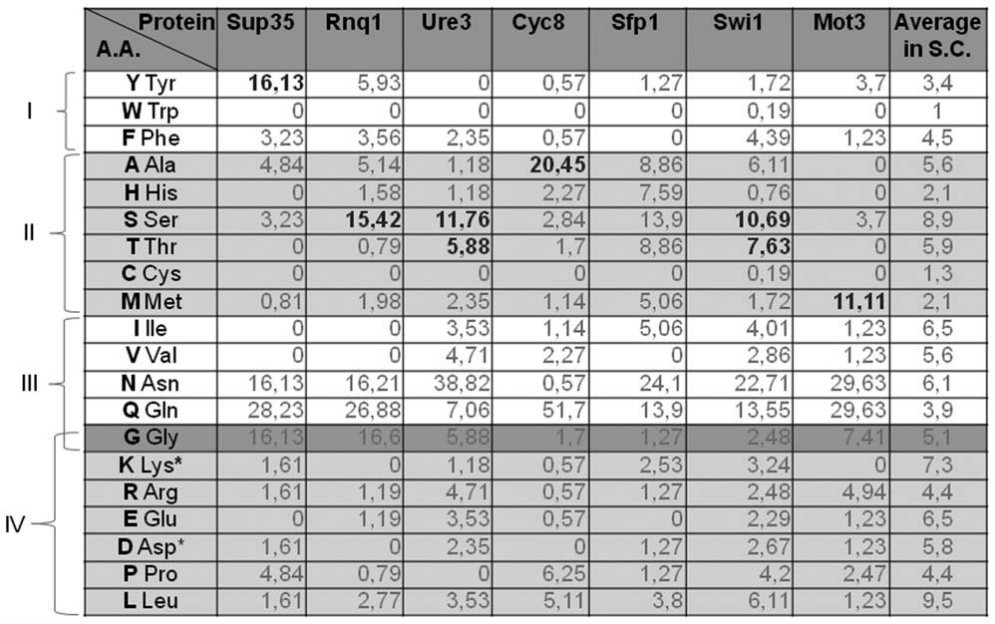
Amino acid content of yeast prion domains. Percentages represent the proportion of the appropriate amino acids in yeast prion domains. The extent of the prion domains were taken from [32]. Fragmentation-promoting amino acids which are abundant in certain prion domains are marked in bold. Numbers on the left represent the group number we assigned to certain residues according to their effect on amyloid formation and fragmentation.

The data in Figure 8 show that some of the amino acids which promote polymer fragmentation in our model system are abundant in the Q/N-rich domains of yeast prions. Interestingly, yeast PrDs are usually enriched in just one of the fragmentation-promoting residues. For example, the Cyc8 PrD has ∼20% alanine, while Ure3 and Rnq1 are rich in serine. Surprisingly, aromatic residues are relatively infrequent in prion domains. Sup35 PrD is the only one abundant in aromatic residues, containing ∼16% tyrosine. Phenylalanine is not abundant, while tryptophan is virtually absent in PrDs.

## Materials and Methods

### Strains and media

PolyQX proteins were produced in the yeast strain 74-D694/ΔS35 which was obtained from 74-D694 (*MATa ura3-52 leu2-3,112 trp1-289 his3-Δ200 ade1-14*) [*PIN* ^+^] by disruption of the chromosomal *SUP35* gene with *TRP1* insertion and introduction of centromeric plasmid pRS315-Sup35C or pRS313-Sup35C [13] encoding the Sup35 C-terminal domain (referred to as Sup35C) to support viability. Deletion of *PRB1* was performed using an integrative plasmid bearing a *PRB1* gene disrupted with a *HIS3* gene. Disruption was confirmed by decreased proteolysis after incubation of samples in SDS-containing sample buffer for 30 minutes in the absence of protease inhibitors. Yeast cells were grown at 30°C in rich YPD (1% yeast extract (Oxoid), 2% peptone (Sigma), 2% glucose), or synthetic (0.67% yeast nitrogen base and 2% glucose supplemented with the required amino acids) medium.

### Plasmid construction

The fusion construct series were prepared using two different strategies. The first was described in [13]. Polyglutamine-encoding DNA constructs were synthesized using three pairs of complementary oligonucleotides. We obtained a series of multicopy yeast plasmids (QX-Sup35MC) expressing fusion proteins with the sequence MSG-(QQQ[X]Q)m-QSQGA-(Sup35MC). Plasmids encoding QY, Q [13], as well as QL, QA, QW and QV were obtained in this fashion. The other strategy was very similar, except that it used a modified cloning vector. Complementary oligonucleotides Bx2B-D: ATTACTTTGGATCCAGTCAAATGG and Bx2B-R: CGCCATTTGACTGGATCCAAAGTAAT were annealed and inserted into plasmid pSBSE [13] digested with with *Sma*I and *Nar*I. This resulted in pBx2B plasmid with two closely spaced *Bst*XI sites for cloning of polyQX-encoding inserts.

We used the following oligonucleotides: Term2-D: AGATCTTATAATGTCGGATC; Term2-R: CTGATCCGACATTATAAGATCTAGTA; Q**X**10-D: AGCAACAAYYYCAACAACAGCAAYYYCAAC; Q**X**10-D+: AGCAACAAYYYCAACAACAGCAAYYYCAACAGTCAA and Q**X**10-R: CTGTTGZZZTTGCTGTTGTTGZZZTTGTTG.. Here, nucleotides YYY complement ZZZ and encode amino acid **X**: F, H, T, S, C, M, N, I, P, R or E. We mixed the Term2-D and Term2-R in a 1∶1 ratio, QX10-D and QX10-R in a 1∶1 ratio and QX10D+ and QX10-R in a 1∶1 ratio. The paired mixtures of oligonucleotides were then annealed and phosphorylated. After that they were mixed in a 1∶20∶1 ratio (Term2-D/Term2-R∶QX10-D/QX10-R∶QX-10D+/QX-10R) and ligated. In this way, polyQX-encoding inserts of different length were assembled, possessing unique sticky ends. These were cloned into the *Bst*XI sites of pBx2B. This strategy turned out to be more efficient, so most of the plasmids were obtained by this method.

The plasmid encoding 85Q-GFP was constructed from the 85Q-MC-encoding plasmid using *in vivo* recombination in yeast. The plasmid YEplac181-NMS35-GFP [30] was digested by *Mlu*I and *Mls*I, mixed with a *Pvu*II and *Ksp*I digested QX-MC-encoding plasmid and this mixture was introduced into yeast by the standard transformation protocol. Plasmids were then purified from yeast cells, introduced into *E. coli*, extracted from *E. coli* and verified by sequencing.

### Preparation of Yeast Cell Lysates

Yeast cultures were grown in liquid medium to A_600_ = 1.5. The cells were harvested; washed in water; and lyzed by glass beads in 25 mM Tris-HCl (pH 7.4), 100 mM NaCl, 1 mM dithiothreitol. To prevent proteolytic degradation, 10 mM phenylmethylsulfonyl fluoride and Complete™ protease inhibitor mixture (Roche Applied Science) were added. Cell debris was removed by centrifugation at 1500 g for 4 min.

### Electrophoresis and blotting

Protein loads were equalized for each gel using a modified biuretic method [31]. Small oligomers of highly fragmented QY, QW and QF proteins were analyzed on SDS-polyacrylamide gels (5% acrylamide, 0.06% bisacrylamide) without boiling of the samples. SDD-AGE was performed according to the standard procedure [10] with modifications used in [32]. We used horizontal 2% agarose gels in Tris acetate/EDTA buffer with 0.1% SDS. Lysates were then mixed with SDD-AGE sample buffer (0.5× Tris acetate/EDTA, 2% SDS, 5% glycerol, and 0.05% Bromophenol blue) and loaded onto the gel. After electrophoresis, the proteins were transferred from gels to nitrocellulose membranes by vacuum-assisted capillary blotting for 5 hours using TBS as transfer buffer. Blots were stained by rabbit primary anti-Sup35NM, Sup35C and anti-GFP (Santa-Cruz) antibodies and anti-rabbit secondary peroxidase-conjugated antibodies (Thermo Scientific). Bound antibody was detected using the SuperSignal West Dura Substrate (Thermo Scientific). It should be noted that SDS in non-boiled samples increases degradation of Sup35 monomers. This can result in the absence of Sup35 monomer bands on semidenaturing detergent-agarose gels. All semidenaturing detergent-agarose gel electrophoresis (SDD-AGE) experiments were repeated at least two times, and typical images are presented.

### Thermal stability assay

Cell lysates were mixed with SDD-AGE sample buffer, transferred to PCR tubes and covered with vaseline oil. The samples were then heated in a Tercik (DNK-Technologii) multi-channel DNA amplifier for 8 minutes at the appropriate temperature and then cooled to 10°C. Cooled samples were then analyzed by SDD-AGE, stained and visualized according to the standard Western blotting protocol. Blots were then quantified densitometrically using ImageJ software.

## Supporting Information

Figure S1 **Polymerization of 101QG and 141QG in a [** ***PIN^+^*** **] background. Lysates of** 74-D694/ΔS35 [*PIN* ^+^] cells producing QX proteins were analyzed by SDD-AGE. (*) shows an overexposed image.
(TIF)

Figure S2 **Polymerization of polyQX proteins in the absence of [** ***PIN*** **^+^] in fresh transformants.** Lysates of 74-D694/ΔS35 Δ*rnq1* cells producing QX proteins of different length were analyzed by SDD-AGE. Staining with anti-S35NM antibodies.
(TIF)

Figure S3 **Intensity plots of the stained SDD-AGE blot images obtained with cells expressing QX proteins in a [** ***PIN*** **^+^] (top panel) and Δ** ***rnq*** **1 (lower panel) background.** Densitometric intensity plots were obtained using ImageJ. The reference lines are derived from the position of DNA fragments from a commercial DNA marker (York Bio) (position visualized by post-run staining with ethidium bromide). The correlation between the mobility of the DNA and protein complexes seems to be constant irrespective of run length (unpublished data). Additional peaks in the intensity profiles correspond to the position of the monomeric protein band. Troughs in the peaks of several panes in the lower panel seem to be artifacts of the electrophoretic run.
(TIF)

Figure S4 **Nonsense-suppression phenotypes of cells producing QX proteins in [** ***PIN*** **^+^] and Δ** ***rnq1*** **backgrounds.** Cells which lost the Sup35C-encoding plasmid were plated onto –Ura YNB plates with a 1/3 amount of adenine to aid the visualization of nonsense-suppressor phenotype. (*) 96QW did not lose the Sup35C encoding plasmid (no spontaneous loss in 300 analyzed clones). See Supplementary Note S1 for details.
(TIF)

Figure S5 **Comparison of 76QY oligomer separation in wild-type and Δ** ***prb1*** **strains.** Lysates of 74-D694/ΔS35 Δ*prb1* and 74-D694/ΔS35 cells were lyzed by SDS-PAGE (large pore 5% gel) without boiling the samples (left panel) and by SDD-AGE (right panel).
(TIF)

Figure S6 **Thermal stability of prion polymers of full-size and truncated Sup35 of an additional strong [** ***PSI*** **^+^] variant.** The lysates of [*PSI* ^+^] yeast cells producing full-sized or truncated Sup35 were analyzed by SDD-AGE. The thermal denaturation curves were derived from densitometric analysis of the stained blot images.
(TIF)

Figure S7 **Thermal stability of 76QY and 120QY polymers.** The lysates of 74-D694/ΔS35 [*PIN* ^+^] cells producing QY76 and QY120 proteins were incubated at different temperatures in the presence of sample buffer containing 2% SDS and analyzed by SDD-AGE. The thermal denaturation curves were obtained by densitometric analysis of the stained blot images.
(TIF)

Supplementary Note S1 **Nonsense suppressor phenotypes of QX-producing cells.**
(DOC)
