# Supplementary figures and images for: The Effects of Amino Acid Composition of Glutamine-Rich Domains on Amyloid Formation and Fragmentation
Source: PLoS One. 2012 Oct 10;7(10):e46458. doi: 10.1371/journal.pone.0046458 (PMC3468588; doi:10.1371/journal.pone.0046458)

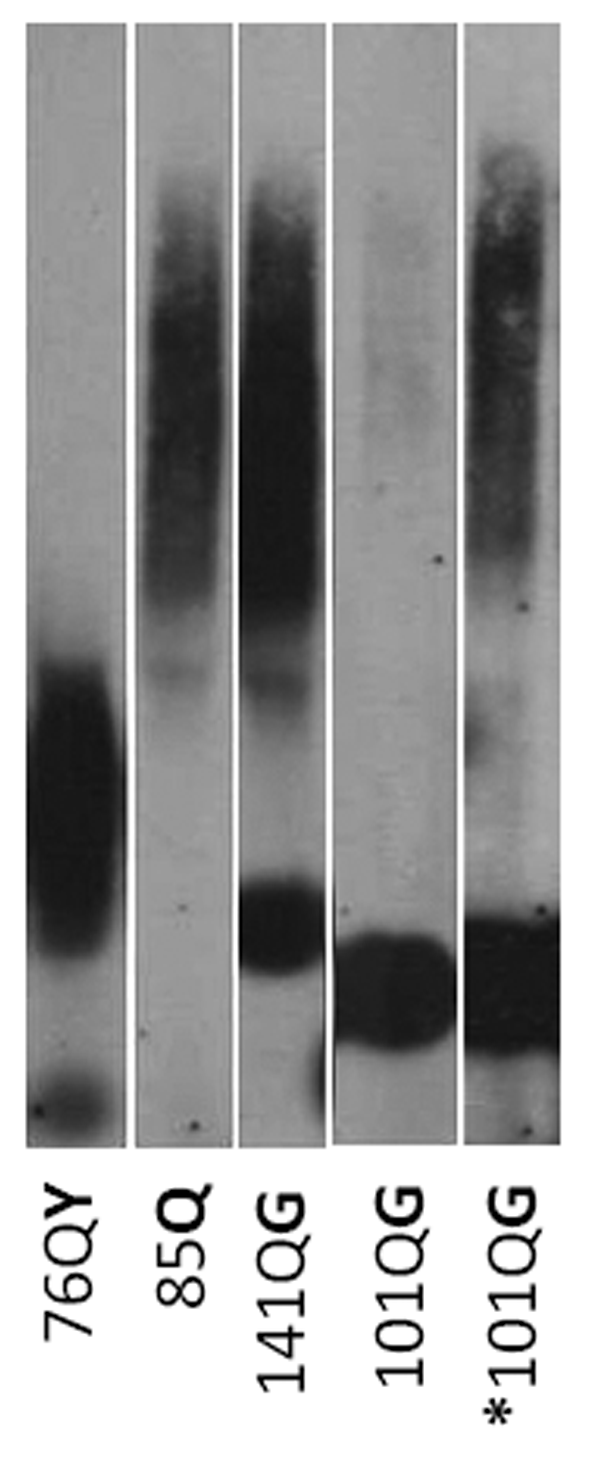

Supplement: Figure S1 — Polymerization of 101QG and 141QG in a [ PIN+ ] background. Lysates of 74-D694/ΔS35 [PIN +] cells producing QX proteins were analyzed by SDD-AGE. (*) shows an overexposed image. (TIF) [file pone.0046458.s001.tif]

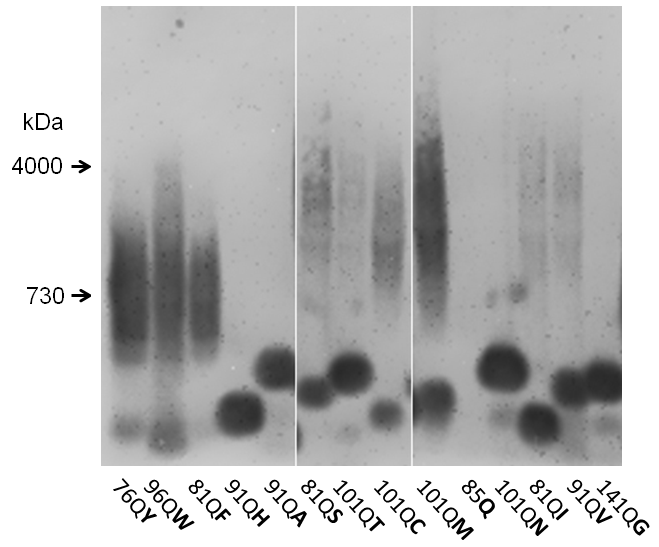

Supplement: Figure S2 — Polymerization of polyQX proteins in the absence of [ PIN +] in fresh transformants. Lysates of 74-D694/ΔS35 Δrnq1 cells producing QX proteins of different length were analyzed by SDD-AGE. Staining with anti-S35NM antibodies. (TIF) [file pone.0046458.s002.tif]

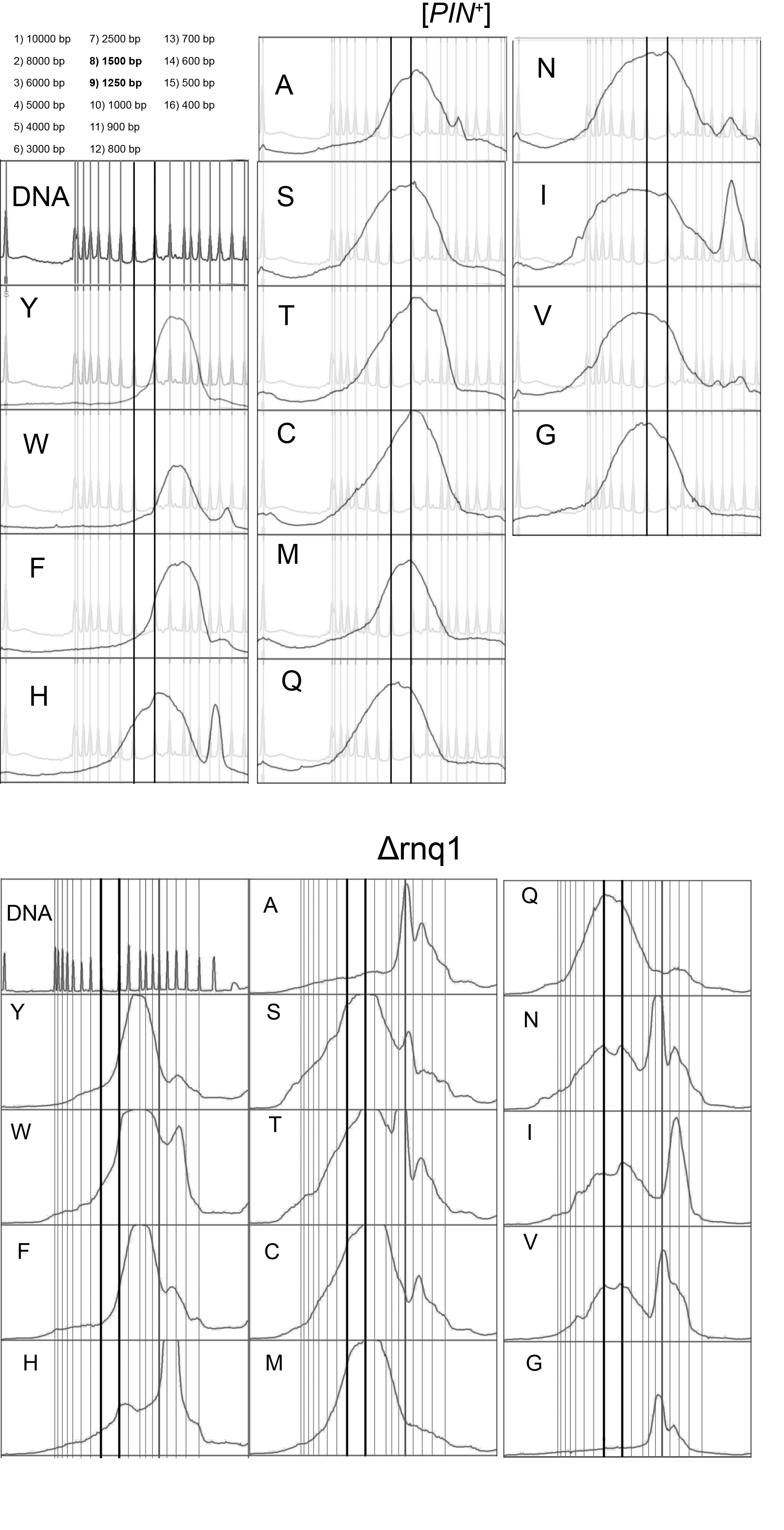

Supplement: Figure S3 — Intensity plots of the stained SDD-AGE blot images obtained with cells expressing QX proteins in a [ PIN +] (top panel) and Δ rnq 1 (lower panel) background. Densitometric intensity plots were obtained using ImageJ. The reference lines are derived from the position of DNA fragments from a commercial DNA marker (York Bio) (position visualized by post-run staining with ethidium bromide). The correlation between the mobility of the DNA and protein complexes seems to be constant irrespective of run length (unpublished data). Additional peaks in the intensity profiles correspond to the position of the monomeric protein band. Troughs in the peaks of several panes in the lower panel seem to be artifacts of the electrophoretic run. (TIF) [file pone.0046458.s003.tif]

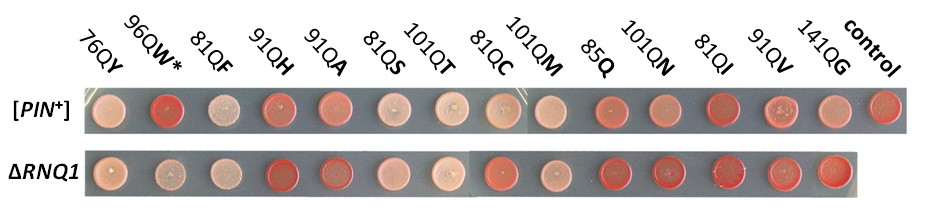

Supplement: Figure S4 — Nonsense-suppression phenotypes of cells producing QX proteins in [ PIN +] and Δ rnq1 backgrounds. Cells which lost the Sup35C-encoding plasmid were plated onto –Ura YNB plates with a 1/3 amount of adenine to aid the visualization of nonsense-suppressor phenotype. (*) 96QW did not lose the Sup35C encoding plasmid (no spontaneous loss in 300 analyzed clones). See Supplementary Note S1 for details. (TIF) [file pone.0046458.s004.tif]

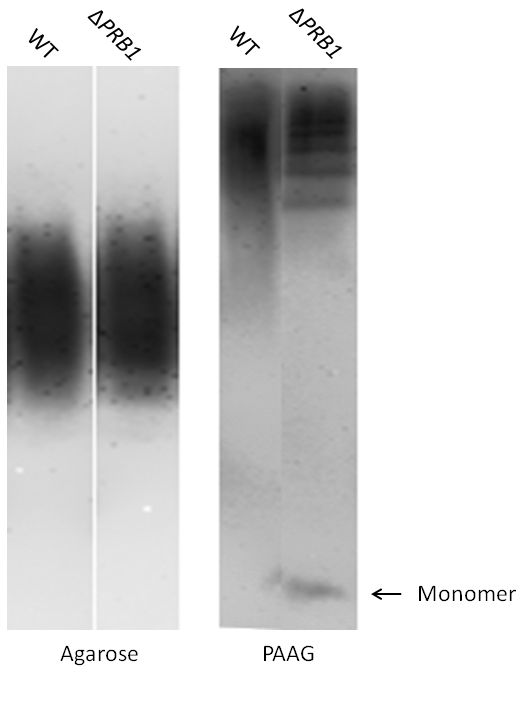

Supplement: Figure S5 — Comparison of 76QY oligomer separation in wild-type and Δ prb1 strains. Lysates of 74-D694/ΔS35 Δprb1 and 74-D694/ΔS35 cells were lyzed by SDS-PAGE (large pore 5% gel) without boiling the samples (left panel) and by SDD-AGE (right panel). (TIF) [file pone.0046458.s005.tif]

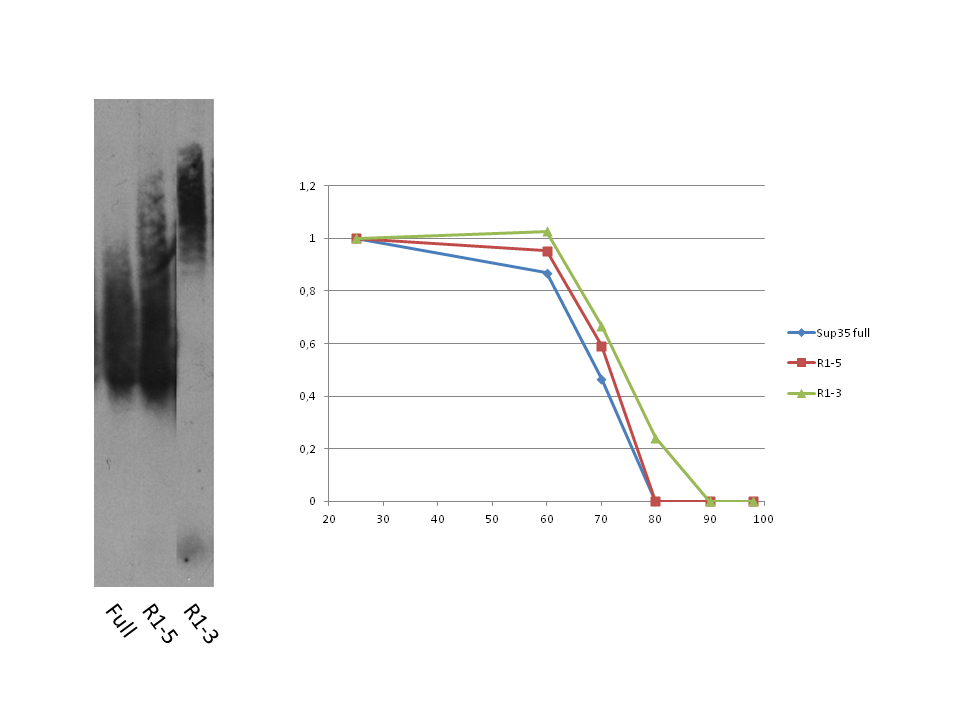

Supplement: Figure S6 — Thermal stability of prion polymers of full-size and truncated Sup35 of an additional strong [ PSI +] variant. The lysates of [PSI +] yeast cells producing full-sized or truncated Sup35 were analyzed by SDD-AGE. The thermal denaturation curves were derived from densitometric analysis of the stained blot images. (TIF) [file pone.0046458.s006.tif]

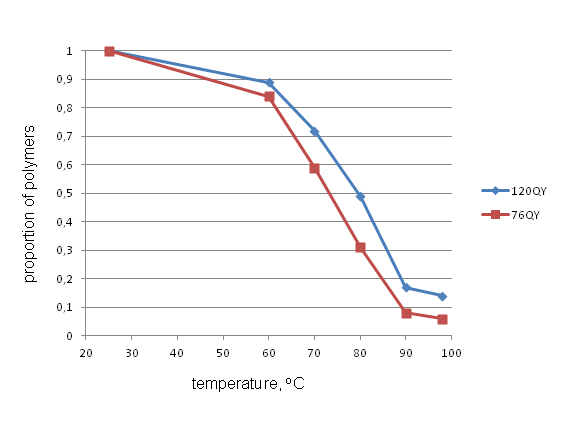

Supplement: Figure S7 — Thermal stability of 76QY and 120QY polymers. The lysates of 74-D694/ΔS35 [PIN +] cells producing QY76 and QY120 proteins were incubated at different temperatures in the presence of sample buffer containing 2% SDS and analyzed by SDD-AGE. The thermal denaturation curves were obtained by densitometric analysis of the stained blot images. (TIF) [file pone.0046458.s007.tif]
