# Supplementary material for: The Effects of Amino Acid Composition of Glutamine-Rich Domains on Amyloid Formation and Fragmentation
Source: PLoS One. 2012 Oct 10;7(10):e46458. doi: 10.1371/journal.pone.0046458 (PMC3468588; doi:10.1371/journal.pone.0046458)
Supplement: Supplementary Note S1 — Nonsense suppressor phenotypes of QX-producing cells. (DOC) [file pone.0046458.s008.doc]

**Supplementary note**

**Nonsense suppressor phenotypes of QX-producing cells**

Nonsense suppressor phenotypes of the cells producing polyQX proteins were scored after the loss of the plasmid encoding the C-domain of Sup35. The 74-D694 cells harbor an *ADE1-14* nonsense mutation, which causes accumulation of red pigment in cells with normal levels of nonsense-suppression. Polymerization decreases the levels of soluble polyQX-Sup35MC proteins, and causes nonsense readthrough which reduces the amount of the red pigment. Thus, whiter colony color indicates higher efficiency of polyQX polymerization. Smaller polymers, which form as a result of fragmentation, have more fiber ends than unfragmented long polymers and thus cause more efficient polymerization. This was observed for strong and weak [*PSI*^+^] variants in (Kryndushkin 2003). For cells producing QX proteins, whiter color also correlated with smaller polyQX polymers, (Fig. S3), though with some exceptions. These could be explained by noting that the suppressor phenotype should depend not only on the polymer size, but also on the rate of polymerization on a single fiber end (Tanaka 2006) and, possibly, the effect of the polyQX domain on the termination activity of soluble polyQX-Sup35 proteins.
